# Rosmarinic Acid-Rich Extracts of Summer Savory (*Satureja hortensis* L.) Protect Jurkat T Cells against Oxidative Stress

**DOI:** 10.1155/2013/456253

**Published:** 2013-11-21

**Authors:** Irakli Chkhikvishvili, Tamar Sanikidze, Nunu Gogia, Tamar Mchedlishvili, Maia Enukidze, Marine Machavariani, Yakov Vinokur, Victor Rodov

**Affiliations:** ^1^Institute of Medical Biotechnology, Tbilisi State Medical University, 33 Vazha Pshavela Ave. 0177, Tbilisi, Georgia; ^2^Department of Postharvest Science of Fresh Produce, Agricultural Research Organization, The Volcani Center, P.O. Box 6, 50250 Bet Dagan, Israel

## Abstract

Summer savory (*Satureja hortensis* L., *Lamiaceae*) is used in several regions of the world as a spice and folk medicine. Anti-inflammatory and cytoprotective effects of *S. hortensis* and of its rosmarinic acid-rich phenolic fraction have been demonstrated in animal trials. However, previous studies of rosmarinic acid in cell models have yielded controversial results. In this study, we investigated the effects of summer savory extracts on H_2_O_2_-challenged human lymphoblastoid Jurkat T cells. LC-MS analysis confirmed the presence of rosmarinic acid and flavonoids such as hesperidin and naringin in the phenolic fraction. Adding 25 or 50 *µ*M of H_2_O_2_ to the cell culture caused oxidative stress, manifested as generation of superoxide and peroxyl radicals, reduced cell viability, G0/G1 arrest, and enhanced apoptosis. This stress was significantly alleviated by the ethanolic and aqueous extracts of *S. hortensis* and by the partially purified rosmarinic acid fraction. The application of an aqueous *S. hortensis* extract doubled the activity of catalase and superoxide dismutase in the cells. The production of IL-2 and IL-10 interleukins was stimulated by H_2_O_2_ and was further enhanced by the addition of the *S. hortensis* extract or rosmarinic acid fraction. The H_2_O_2_-challenged Jurkat cells may serve as a model for investigating cellular mechanisms of cytoprotective phytonutrient effects.

## 1. Introduction

Summer savory (*Satureja hortensis* L.) is an herb of the *Lamiaceae* family that is used in cooking and folk medicine in several regions of the world [1]. In Georgia, dried and ground summer savory (local name *kondari*) is one of the most popular spices, used either on its own or as an ingredient in spice blends. In addition, from ancient times, it has been known locally as an antimicrobial folk remedy for gastrointestinal problems [2]. Indigenous landraces of summer savory are cultivated in Georgia [3]. 

The leaves of summer savory are rich in phenolic compounds, particularly rosmarinic acid and flavonoids, which account for the high antioxidant capacity of these leaves [4, 5]. In our previous study of Georgian spices, we found that *kondari* had one of the highest total phenolic content levels and one of the highest hydrophilic antioxidant capacity levels [6]. Rosmarinic (**α**-O-caffeoyl-3,4-dihydroxy-phenyl lactic) acid was found to be the major compound in ethanolic extracts of summer savory and some other *Lamiaceae* herbs [4]. Rosmarinic acid is a phenylpropanoid derivative that is the second most common ester of caffeic acid in the plant kingdom.

Animal studies have revealed anti-inflammatory activity of *S. hortensis* extract and its polyphenolic fraction, in particular [7, 8]. This activity might be associated, at least partially, with rosmarinic acid, whose antiinflammatory and antiallergic properties have been demonstrated in animal and human trials [9, 10]. Osakabe et al. [10] suggested that the antiallergic effect of rosmarinic acid might be due to two independent mechanisms: the scavenging of reactive oxygen species and the modulation of the inflammatory response. For example, the nephroprotective effect of rosmarinic acid was associated with improved antioxidant potency, including enhanced glutathione content and activity of antioxidant enzymes [11].

However, the cellular mechanisms by which rosmarinic acid exerts its anti-inflammatory effects are not fully understood and demand further investigation. The human lymphoblastoid T-cell Jurkat line, a constitutive producer of the potent T-cell growth factor interleukin 2 (IL-2), is a popular model for the study of immune signaling [12]. Jurkat cells can imitate both healthy and inflammatory T-cells in their response to oxidative metabolites, such as hydrogen peroxide [13]. Therefore, investigating the effect of *S. hortensis* extract on the proliferation and activity of  T-cells may contribute to our understanding of the mechanism(s) of its anti-inflammatory and cytoprotective effects. Although H_2_O_2_ plays an important role in antigen-dependent lymphocyte activation [14], excessive production of H_2_O_2_ induces oxidative stress and impairs T-cell activity, leading to chronic inflammation and cell death.

The response of Jurkat cells to H_2_O_2_ is dose-dependent. Reversible oxidative changes that can be repaired by cellular antioxidant system occur at a H_2_O_2_ concentration of 20 *μ*M, and the first signs of apoptosis are noted at 50 *μ*M H_2_O_2_ [15]. Relatively high bolus doses of H_2_O_2_ (150 *μ*M) induce apoptosis in Jurkat cells, but the continuous presence of a lower concentration of H_2_O_2_ (2 *μ*M) inhibits the apoptotic process [16]. Both apoptosis and necrosis were observed in the Jurkat cells exposed to 100 *μ*M H_2_O_2_ [17], while necrosis was far more common at 500 *μ*M H_2_O_2_ [18]. Despite its well-documented cytoprotective activity in animal trials, concentrations of up to 150 *μ*M of rosmarinic acid failed to prevent the H_2_O_2_-mediated apoptosis of Jurkat cells and showed no antioxidant properties [19]. Moreover, even in the absence of exogenous hydrogen peroxide, rosmarinic acid was reported to induce the apoptosis of Jurkat cells [19, 20].

The discrepancy between the prooxidant behavior of rosmarinic acid toward Jurkat cells that has been observed in previous studies and its well-known antioxidant and anti-inflammatory properties hamper the use of Jurkat cells as a model for investigating the mode of action of this phytonutrient. In the present work, we reexamined the effects of summer savory extracts and thier rosmarinic acid-rich phenolic fraction on H_2_O_2_-challenged Jurkat cells.

## 2. Materials and Methods

### 2.1. Plant Material

Plants of a local Georgian landrace of *S. hortensis* were grown in an experimental plot near Tbilisi from seeds purchased from commercial supplier. The plants were harvested at their vegetative state (55 days after seed germination), the phenological stage characterized by the highest phenolic compound content, highest flavonoid content, and greatest antioxidant activity (I. Chkhikvishvili, unpublished data). The collected plant material was air-dried in the shade at 25–30°C. The dried matter was stored in a closed glass container in a cool, dry place.

### 2.2. Extraction and Purification

The dried plant material (1 g samples) was sequentially extracted with chloroform, ethyl acetate, and ethanol at a 1 : 5 w/v ratio of plant material to solvent; the duration of each extraction step was 24 h. The residue was extracted with water by steeping for 20 min at 90°C and subsequent gradual cooling down to room temperature. Direct application of this water extraction technique to the dried plant material produced a “total aqueous extract.” The solvents were removed by evaporation under vacuum at a temperature below 40°C, and the extracts were stored at −80°C until use. For the purification of the phenolic fraction, the total aqueous extract was percolated through a polyamide column. The column was washed with water and the purified fraction was eluted with 96% ethanol.

### 2.3. Liquid Chromatography-Mass Spectrometry (LC-MS) Analysis

The samples of purified phenolic fraction were dissolved in HPLC-grade methanol and filtered through a Millex-HV Durapore (PVDF) membrane (0.22 *μ*m) before being injected into the LC-MS instrument. Mass spectral analyses were carried out using the Ultraperformance LC-Quadruple Time of Flight (UPLC-QTOF) instrument (Waters Premier QTOF, Milford, MA, USA), with the UPLC column connected online to a PDA detector (Waters Acquity), and then to an MS detector equipped with an electrospray ion (ESI) source (used in ESI-negative mode). Separation was performed on a 2.1 × 50 mm i.d., 1.7 *μ*m UPLC BEH C18 column (Waters Acquity). 

The chromatographic and MS parameters were as follows: the mobile phase consisted of 0.1% formic acid in water (phase A) and 0.1% formic acid in acetonitrile (phase B). The linear gradient program was as follows: 100% to 95% A over 0.1 min, 95% to 5% A over 9.7 min, held at 5% A over 3.2 min, and then returned to the initial conditions (95% A) in 4.2 min. The flow rate was 0.3 mL min^−1^ and the column was kept at 35°C. Masses of the eluted compounds were detected with a QTOF Premier MS instrument. The UPLC-MS runs were carried out at the following settings: capillary voltage of 2.8 kV, cone voltage of 30 eV, and collision energy of 5 eV. Argon was used as the collision gas. The *m/z* range was 70 to 1,000 D. The MS system was calibrated using sodium formate and Leu-enkephalin was used as the lock mass. The MassLynx software version 4.1 (Waters) was used to control the instrument and calculate accurate masses.

### 2.4. Cell Culture and Experimental Design

The human T-cell leukemia lymphoblastoid Jurkat cells (DSMZ ACC 282) were obtained from the Deutsche Sammlung von Mikroorganismen und Zellkulturen (DSMZ, Braunschweig, Germany). The cells were grown in suspension culture at 37°C under 5% humidified CO_2_ in bioactive medium RPMI 1640 (Gibco, Grand Island, NY, USA) containing inactivated embryonic bovine serum (Sigma, St. Louis, MO, USA), L-glutamine (4 mM), penicillin (100 U mL^−1^), and streptomycin (100 U mL^−1^). The experiments were carried out at cell densities of 0.3 to 0.6 × 10^6^ cells mL^−1^. In order to imitate the oxidative stress conditions, H_2_O_2_ (Sigma) was added to the Jurkat culture to reach the concentrations of 25 and 50 *μ*M, corresponding to low and intermediate stress severity, respectively [15]. In the unstressed control treatment, water was added to the samples instead of H_2_O_2_. The *S. hortensis* extracts were added to the cultures at a rate of 2 mg mL^−1^ as the H_2_O_2_ was added.

In a separate trial, the effect of cell pretreatment with *S. hortensis* extract on their response to subsequent H_2_O_2_ oxidative stress was investigated. Cell suspensions (2 × 10^6^ cells mL^−1^) were incubated with *S. hortensis* rosmarinic acid fraction as described above. After the incubation period, the cells were harvested by centrifugation at 1500 g for 5 minutes, washed, resuspended in fresh medium, and exposed to H_2_O_2_. Cellular responses to oxidative stress were evaluated by free radicals generation and cell viability as described below.

### 2.5. Hydrogen Peroxide Scavenging Capacity

The ability of *S. hortensis* extracts to scavenge hydrogen peroxide in the absence of cells was tested in order to check possible contribution of this abiotic H_2_O_2_ decomposition to experimental results. The H_2_O_2_-scavenging capacity of extracts was tested as described by Ruch et al. [21]. A solution of hydrogen peroxide 50 *μ*M was prepared in phosphate buffer (pH 7.4). Phenolic extracts (2 mg mL^−1^) in distilled water and 50 *μ*M hydrogen peroxide solution were added to incubation system comprising bioactive medium RPMI 1640 (GIBSO) with inactivated embryonic bovine serum (Sigma), L-glutamine (4 mM), penicillin (100 U mL^−1^), and streptomycin (100 U mL^−1^). Absorbance of hydrogen peroxide at 230 nm was determined 10 minutes later against a blank solution containing the incubation medium with hydrogen peroxide. The percentage of hydrogen peroxide scavenging by *S. hortensis* extracts was calculated. The trial revealed a 17% reduction of H_2_O_2_ concentration due to the interaction with *S. hortensis* rosmarinic acid fraction. 

### 2.6. Electron Paramagnetic Resonance (EPR) Spectroscopy

The effect of *S. hortensis* extracts on the generation of free radicals in H_2_O_2_-challenged and unchallenged cells was studied using the electron paramagnetic resonance (EPR) method. EPR spectra were registered on a radiospectrometer, RE 1307 (EPSI, Chernogolovka, Russia). Peroxyl radicals were detected with spin-trap *α*-phenyl-tertbutylnitrone (PBN; Sigma) (50 mM on 0.6 × 10^6^ cells in 0.5 mL medium) at room temperature at microwave power (20 mV). Superoxide radicals were detected with a spin-trap 5,5 dimethyl-I-pyrrolyn-IV-oxide (DMPO) (Sigma) (50 mM on 0.6 × 10^6^ cells in 0.5 mL medium) at room temperature at microwave power (20 mV).

### 2.7. Cell Viability and Proliferation

The viability of the cells was determined using the MTT cell proliferation assay. Cell suspensions (2 × 10^6^ cells mL^−1^) were incubated with H_2_O_2_ and *S. hortensis* preparations as described above. After the incubation period, the cells were harvested by centrifugation at 1500 g for 5 minutes, washed, and resuspended in fresh medium. The 8 mg mL^−1^ solution of 3-(4,5-dimethylthiazol-2)-2,5-diphenyltetrazolium bromide (MTT) (Sigma) in buffer (140 mM NaCl, 5 mM HEPES, pH 7.4) was added to the cell suspension at a rate of 30 *μ*L per 100 *μ*L suspension and the mixture was incubated for 4 h at 37°C in a 5% CO_2_ atmosphere. After this incubation, the supernatant was carefully removed and the colored formazan crystals produced from the MTT were dissolved in 100 *μ*L of dimethyl sulfoxide (DMSO). The absorption values of the solutions were measured at 570 nm. The distribution of the Jurkat cells among the different cell-cycle phases was studied using flow cytometry. Mitochondrial transmembrane potential (ΔΨ) in the cell culture was determined by flow cytometry using the lipophilic cation test 3,3′-dihexyloxacarbocyanine iodide (DiOC_6_) described by Zamzami et al. [22]. 

### 2.8. Antioxidant Enzymes

Jurkat cell extract was prepared by centrifuging the cell suspensions at 500 g and then homogenizing the cellular precipitate in a lysis buffer (pH 7.9) that was comprised of 1.5 mM MgCl_2_, 10 mM KCl, 1 mM dithiothreitol, 1 *μ*g mL^−1^ leupeptin, 1 *μ*g mL^−1^ aprotinin, and 10 mM HEPES. The volume of the buffer was twice the volume of the precipitate. Lysis of the cells was performed by passing the suspension through a 27-gauge needle 10 times. The obtained homogenate was centrifuged for 20 min at 10,000 g. The supernatant was used to determine the levels of enzyme activity. Catalase (EC 1.11.1.6) activity was measured spectrophotometrically as the decomposition of H_2_O_2_ at 240 nm [23]. One unit of catalase activity was defined as the amount of enzyme decomposing 1 *μ*mol of H_2_O_2_ per minute. The superoxide dismutase (SOD; EC 1.15.1.1) was assayed using NADPH and phenazine methosulfate (PMS) reagents for the reduction of nitroblue tetrazolium salt (NBT) into blue-colored formazon measured spectrophotometrically at 560 nm [24]. One unit of SOD activity was defined as the amount of enzyme oxidizing 1 nmol NADPH per minute. The activity of both enzymes was expressed in terms of units per mg of protein. A total protein micro Lowry kit (Sigma) was used to determine the protein content.

### 2.9. Interleukin Analysis

Jurkat cells were prestimulated by incubation with 50 *μ*g/mL phytohemagglutinin (PHA) at 37°C for 5 min and cultured for 24 h with nonstimulated Jurkat cells (40% stimulated and 60% non-stimulated cells). The pro- and anti-inflammatory cytokines IL-2 and IL-10 were assayed using ELISA kits (Bender Medsystems, Vienna, Austria) and the Multiscan microplate reader (LabSystem, Helsinki, Finland). 

### 2.10. Statistics

The trials were performed in five replications. The statistical analysis of the obtained results, including calculation of means and standard deviations, was conducted using the IBM SPSS Statistics program. The statistical significance of the differences between the means was analyzed by pair-wise comparison of treatment results with nontreated control using Student's *t*-test at *P* < 0.05.

## 3. Results

### 3.1. Analysis of *S. hortensis* Extracts

**Figure 1:**
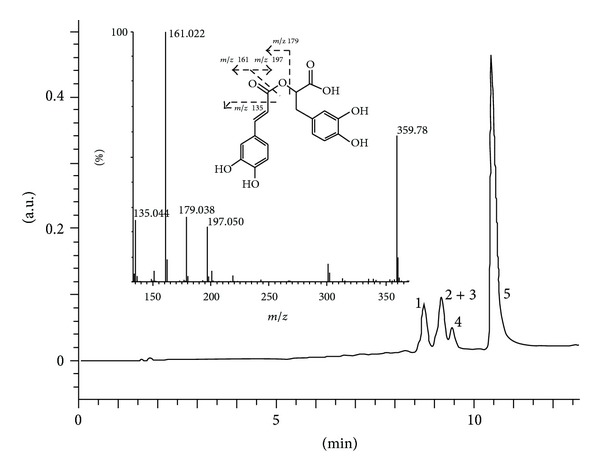
HPLC chromatogram of the *S. hortensis* rosmarinic acid fraction. The peak 5 represents rosmarinic acid and the peak 2 + 3 partially separated naringin and hesperidin. The peaks 1 and 4 were tentatively identified as rutin and apigenin-7-glucoside, respectively. Insert: mass-spectrum of the rosmarinic acid and its fragmentation scheme.

HPLC analysis revealed a number of phenolic compounds in the ethanolic extract of *S. hortensis*, rosmarinic and ferulic acids being the major compounds. In addition, a number of phenolic acids (caffeic, *p*-coumaric), flavonoid aglycones (catechin, epicatechin, luteolin, apigenin), and glycosides (rutin, hesperidin, apigenin-7-glucoside) were tentatively identified in the ethanolic extract. Partial purification of the rosmarinic acid provided a fraction comprising four major peaks. The tentative identification of the rosmarinic acid as the most abundant component of the fraction was based on its UV absorption spectrum and retention time as compared with those of the authentic standard sample. The identity was confirmed by LC-MS based on the presence of a deprotonated molecular ion [M−H]^−^ at *m/z* 359 and characteristic fragment ions at* m/z* 123,* m/z* 135, *m/z* 161, *m/z* 179, and *m/z* 197, in accordance with data in the literature [25, 26] and fragmentation scheme (Figure 1). Two flavonoid glycosides were identified by LC-MS through comparisons with standard samples as hesperidin based on a [M−H]^−^ at *m/z* 609, a characteristic hesperetin fragment ion at *m/z* 301, naringin based on [M−H]^−^ at *m/z* 579, and a characteristic naringenin fragment ion at *m/z* 271. In addition, two more flavonoid glycosides were tentatively identified in the fraction as rutin and apigenin-7-glucoside.

*H*_*2*_ *O*_*2*_ *-Induced Oxidative Stress as Affected by S. hortensis Extracts.* The addition of 25 or 50 *μ*M of hydrogen peroxide caused oxidative stress in the Jurkat cells, which was manifested as the generation of superoxide and peroxyl radicals that could be detected by EPR spectroscopy. The amount of radicals formed depended on the concentration of H_2_O_2_; no radicals were detected in the absence of hydrogen peroxide (Table 1).

**Figure 2:**
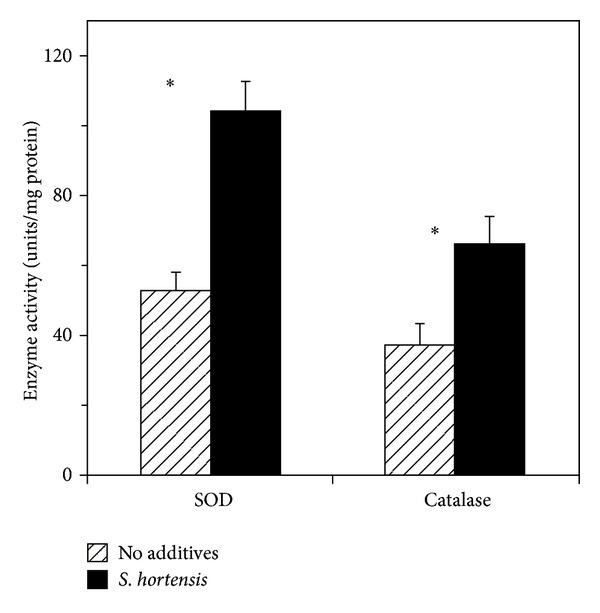
Effect of the total aqueous *S. hortensis* extract on the activities of superoxide dismutase (SOD) and catalase in Jurkat cells. Error bars represent standard deviations of five replications. Bars marked with an asterisk are significantly different from the control at *P* ≤ 0.05, according to Student's *t*-test.

Chloroform and ethyl acetate extracts of *S. hortensis* had only limited effects on the oxidative state of the cells, slightly reducing the amount of radicals detected at higher hydrogen peroxide concentrations. On the other hand, considerable alleviation of the oxidative stress and almost complete elimination of the radicals were observed in the presence of the ethanolic *E. hortensis* extract. Significant antioxidant effects were also associated with the aqueous extract and with the partially purified rosmarinic acid fraction, although the efficacy of the latter preparation was markedly lower than that of the crude ethanolic extract. In line with these findings, the total aqueous extract of *S. hortensis *doubled the activity of the antioxidant enzymes catalase and superoxide dismutase in the Jurkat cells, even in the absence of exogenous hydrogen peroxide (Figure 2). 

### 3.2. Effects on Jurkat Cell Viability

In the absence of any exogenous H_2_O_2_ challenge, adding ethanolic *S. hortensis* extract or the purified phenolic fraction to Jurkat cells slightly improved their viability. Other *S. hortensis* extracts had no significant effects on the viability of unstressed Jurkat cells, as measured by the MTT test (Table 2).

Hydrogen peroxide-induced oxidative stress reduced the viability of Jurkat cells in a dose-dependent manner. This hydrogen peroxide effect was alleviated by the application of ethanolic and aqueous extracts of *S. hortensis* and by the phenolic fraction. The aqueous *S. hortensis* extract was the most effective for restoring cell viability to the level observed in the unstressed control culture (Table 2).

### 3.3. Effect of Pretreatment of Jurkat Cells with *S. hortensis* Extract on Subsequent Cellular Sensitivity to Oxidative Stress

The data presented in Table 3 demonstrate that pretreatment of Jurkat cells with the rosmarinic acid fraction significantly alleviated the oxidative stress incurred to cells by subsequent exposure to hydrogen peroxide, as expressed by free radical generation and decline in cell viability. This alleviation could not be attributed to the peroxide-scavenging activity of the extracts because no direct contact of the extracts with the peroxide took place in that case. In addition, the direct peroxide-scavenging capacity of the rosmarinic acid fraction did not exceed 17%, so that its contribution to the cell protection was rather limited. 

### 3.4. Effects on the Cell Cycle

Oxidative stress changed the cell-cycle phase distribution of the Jurkat cells, restricting cell proliferation and increasing the relative proportions of G0/G1 cells (the G0/G1 arrest) and apoptotic cells among the total cell population. These trends were alleviated by the addition of the ethanolic *S. hortensis* extract, so that the amount of apoptotic cells in that treatment was not significantly different from that observed in the unstressed control (Table 4). Adding the *S. hortensis* extract alone, without hydrogen peroxide, had no significant effect on the cell-cycle phase distribution of the Jurkat cells (data not shown). The alleviation of H_2_O_2_-induced apoptosis by the ethanolic *S. hortensis* extract and by the partially purified rosmarinic acid fraction was also evident from the index of mitochondrial transmembrane potential determined by flow cytometry (Table 5).

### 3.5. Interleukin Production

The production of both IL-2 and IL-10 interleukins by Jurkat cells was stimulated by hydrogen peroxide and further enhanced by the addition of the *S. hortensis* extract and its phenolic fraction (Table 6).

## 4. Discussion

Our study has confirmed that rosmarinic acid is an abundant phenylpropanoid compound in summer savory. To the best of our knowledge, hesperidin and naringin have not been previously reported in *S. hortensis*, but they have been found in other *Satureja* species [27] and in other genera of this family, such as *Mentha* [25]. 

The present research has demonstrated for the first time that *S. hortensis* and its rosmarinic acid-rich fraction can protect Jurkat cells from oxidative stress caused by hydrogen peroxide. These findings are in line with the antioxidant, cytoprotective, and anti-inflammatory activities of *S. hortensis* [7] and rosmarinic acid [9, 10] that have been observed *in vivo* in animals and humans. Similar protective antioxidant properties were exhibited by *S. hortensis* extracts when applied to H_2_O_2_-stressed lymphocytes isolated from blood taken from healthy rats [28]. In cell cultures, rosmarinic acid protected human neuronal cells against hydrogen peroxide-induced apoptosis [29] and inhibited in a dose-dependent manner the formation of reactive oxygen and nitrogen species in RAW264.7 macrophages stimulated with lipopolysaccharide or phorbol 12-myristate 13-acetate [30].

On the other hand, in a previous study, rosmarinic acid failed to protect Jurkat cells from H_2_O_2_-mediated oxidative damage and actually induced their apoptosis [19, 20]. Such prooxidant cytotoxic reactions in cell cultures are associated with the generation of H_2_O_2_ through the interaction of phenolic compounds with culture media ingredients (e.g., transient metals) and can, therefore, be considered artifacts [31, 32]. Inclusion of catalase or metmyoglobin in the growth medium negates these reactions and allows the realization of the cytoprotective antioxidant potential of phenolic compounds [31]. 

One possible explanation for the apparent discrepancy between our results and those of Kolettas et al. [19] might be that the high dose of antioxidant materials used in our study could overcome the influence of H_2_O_2_, either added exogenously or generated in cell cultures with participation of transient metals. Indeed, in a metal-catalyst system, most phenolic compounds exhibited pro-oxidant effects at low doses and shifted to antioxidant activity at higher concentrations [33]. Furthermore, it was shown recently that high doses (2-3 mM) of caffeic acid and other phenylpropanoids protected Jurkat cells from H_2_O_2_-induced DNA damage by chelating intracellular labile iron [34]. The presence of the potent flavonoid antioxidants in the phenolic fraction, in addition to rosmarinic acid, might further strengthen its antioxidant capacity. Enhancement of the activity of the antioxidant enzymes by *S. hortensis* (Figure 2) might also contribute to the neutralization of hydrogen peroxide. Catalase and SOD play important roles in the control of oxidative stress and apoptosis in Jurkat cells [35]. Similar to our findings, an aqueous extract of another rosmarinic acid-containing *Lamiaceae* herb, *Perilla frutescens*, was shown to upregulate the mRNA and protein expression of these antioxidant enzymes in cultured human vein endothelial cells [36].

Another noteworthy phenomenon observed in this work was a parallel increase in the levels of the IL-2 and IL-10 interleukins. Robust production of IL-2 is the major trait of the Jurkat cell line [12]. There is a synergistic interaction between these two interleukins during the immune response [37]. Anti-inflammatory factors such as IL-10 may be released in order to balance the dramatic increase in proinflammatory cytokines in stressful situations, and thereby control the magnitude and duration of the inflammatory response [38]. Interestingly, adding antioxidant-rich plant materials to the diets of animals enduring proinflammatory conditions has been shown to increase the level of IL-10 [39] or the levels of both IL-2 and IL-10 [40] in parallel with a decrease in the levels of pro-inflammatory factors, such as IL-6, TNF-*α*, and IL-1*β*. In addition, these dietary interventions preserved normal antioxidant enzyme activity, inhibited lipid peroxidation, and increased the HDL levels in the treated animals, resulting in the alleviation of disorders and enhanced immunity. Rosmarinic acid increased the secretion of IL-10 in a lipopolysaccharide-stimulated macrophage model [41].

Addition of the *S. hortensis* extract or its phenolic fraction restored the viability and proliferation of H_2_O_2_-challenged Jurkat cells, alleviated the G0/G1 arrest, and controlled the apoptosis of these cells. Altogether, these phenomena were in line with the general scheme of cellular response to oxidative stress, implying that low doses of reactive oxygen species promote cell proliferation, intermediate doses result in growth arrest, and severe oxidative stress ultimately causes cell death via apoptotic or necrotic mechanisms [42]. Apparently, the addition of *S. hortensis* extracts alleviated the oxidative stress exerted on the cells by hydrogen peroxide. These effects may be attributed to the direct radical-scavenging activity of rosmarinic acid and other phenolic compounds, as well as to indirect mechanisms such as the enhancement of antioxidant enzymes and the release of anti-inflammatory signaling molecules, such as IL-10.

## 5. Conclusions

The present research has demonstrated that rosmarinic acid-rich extract of *S. hortensis* can protect Jurkat cells from oxidative stress caused by hydrogen peroxide. These findings are in line with the antioxidant, cytoprotective, and anti-inflammatory activities of rosmarinic acid that have been observed in animals and humans. Therefore, the H_2_O_2_-challenged Jurkat cells may serve a model for investigating cellular mechanisms of cytoprotective effects of phytonutrients. It should be kept in mind, however, that these results were achieved with a rather high concentration of rosmarinic acid that supposedly could overcome the culture-associated artifacts. Further research is needed, in order to optimize the experimental system. 

## Figures and Tables

**Table 1:** Effects of *S. hortensis* extracts and of the partially purified rosmarinic-acid fraction on the generation of superoxide (O_2_^−^) and peroxyl (LOO^∙^) radicals in Jurkat cells subjected to hydrogen peroxide-induced oxidative stress.

	Hydrogen peroxide concentration, *μ*M					
	0		25		50	
	EPR signal intensity, arbitrary units					
	O_2_^−^	LOO^∙^	O_2_^−^	LOO^∙^	O_2_^−^	LOO^∙^
No additives (control)	0	0	2.4 ± 0.2	3.0 ± 0.2	3.1 ± 0.1	3.8 ± 0.3
Chloroform extract	0	0	2.0 ± 0.2	3.2 ± 0.2	2.1 ± 0.2*	2.8 ± 0.3*
Ethyl acetate extract	0	0	2.0 ± 0.2	3.2 ± 0.2	2.1 ± 0.2*	2.8 ± 0.3*
Ethanolic extract	0	0	0*	0*	0.1 ± 0.1*	0*
Aqueous extract	0	0	0*	0.3 ± 0.1*	0.1 ± 0.1*	0.5 ± 0.1*
Rosmarinic acid fraction	0	0	1.0 ± 0.2*	2.2 ± 0.2*	1.1 ± 0.2*	1.8 ± 0.3*

Values represent averages of five replications ± standard deviations. Values marked with the asterisk are significantly different from the control in the same column at *P* ≤ 0.05, according to Student's *t*-test.

**Table 2:** Effects of *S. hortensis* extracts on the viability of Jurkat cells in the presence or absence of hydrogen peroxide.

	Hydrogen peroxide concentration, *μ*M		
	0	25	50
	MTT test results, A_570_		
No additives (control)	0.69 ± 0.02	0.36 ± 0.01	0.22 ± 0.01
Chloroform extract	0.63 ± 0.02	0.37 ± 0.01	0.24 ± 0.03
Ethyl acetate extract	0.58 ± 0.05	0.47 ± 0.03	0.42 ± 0.03*
Ethanolic extract	0.74 ± 0.03*	0.56 ± 0.05*	0.46 ± 0.03*
Aqueous extract	0.61 ± 0.01	0.68 ± 0.04*	0.67 ± 0.02*
Rosmarinic acid fraction	0.75 ± 0.04*	0.62 ± 0.03*	0.42 ± 0.04*

Values represent averages of five replications ± standard deviations. Values marked with the asterisk are significantly different from the control in the same column at *P* ≤ 0.05, according to Student's *t*-test.

**Table 3:** Effect of pretreatment of the Jurkat cells with partially purified *S. hortensis* rosmarinic acid fraction on the cellular response to subsequent hydrogen peroxide-induced oxidative stress.

	Hydrogen peroxide concentration, *μ*M		
	0	25	50
	Peroxyl radicals generation, EPR signal intensity (arbitrary units)		
Nontreated control	0	3.0 ± 0.2	3.8 ± 0.3
Rosmarinic acid fraction	0	1.9 ± 0.2*	2.1 ± 0.3*

	Cell viability (MTT test results, A_570_)		
Nontreated control	0.69 ± 0.02	0.36 ± 0.01	0.22 ± 0.01
Rosmarinic acid fraction	0.75 ± 0.04*	0.59 ± 0.04*	0.37 ± 0.03*

Values represent averages of five replications ± standard deviations. Values marked with the asterisk are significantly different from the control in the same column at *P* ≤ 0.05, according to Student's *t*-test.

**Table 4:** Effects of hydrogen peroxide and of the ethanolic *S. hortensis *extract on the cell-cycle phase distribution of Jurkat cells.

	Cell-cycle phases, %			
	G0/G1	S	G2/M	G0/Apoptosis
No additives (control)	23.8 ± 3.4	54.5 ± 3.3	19.0 ± 2.9	2.7 ± 3.6
H_2_O_2_ 25 *μ*M	42.3 ± 3.3*	36.7 ± 3.4*	12.5 ± 1.7*	8.5 ± 1.9*
H_2_O_2_ 25 *μ*M + *S. hortensis* (ethanolic extract)	37.5 ± 2.5*	43.0 ± 3.3*	16.0 ± 3.4	3.5 ± 1.3

Values represent averages of five replications ± standard deviations. Values marked with the asterisk are significantly different from the control in the same column at *P* ≤ 0.05, according to Student's *t*-test.

**Table 5:** Effects of ethanolic *S. hortensis* extract and of the partially purified rosmarinic-acid fraction on the incidence of apoptosis in Jurkat cells in the presence of hydrogen peroxide.

	Cell counts		*K* ratio*
	Healthy	Apoptotic	
No additives (control)	212	8	26.5
H_2_O_2_ 25 *μ*M	268	3519	0.08
H_2_O_2_ 25 *μ*M + *S. hortensis* (ethanolic extract)	2090	539	3.9
H_2_O_2_ 25 *μ*M + rosmarinic acid fraction	1211	108	11.2

*K-ratio of healthy to apoptotic Jurkat cells.

**Table 6:** Effects of the ethanolic *S. hortensis* extract and of the partially purified rosmarinic-acid fraction on the production of interleukins by Jurkat cells in the presence of hydrogen peroxide.

	IL-2, pg mL^−1^	IL-10, pg mL^−1^	IL-2/IL-10
No additives (control)	0.90 ± 0.05	3.21 ± 0.04	0.28 ± 0.04
H_2_O_2_ 25 *μ*M	2.61 ± 0.04*	6.80 ± 0.05*	0.38 ± 0.01
H_2_O_2_ 25 *μ*M + *S. hortensis* (ethanolic extract)	15.30 ± 0.04*	20.01 ± 0.08*	0.76 ± 0.07
H_2_O_2_ 25 *μ*M + *S. hortensis* rosmarinic acid fraction	20.80 ± 0.07*	38.40 ± 0.06*	0.54 ± 0.07

Values represent averages of five replications ± standard deviations. Values marked with the asterisk are significantly different from the control in the same column at *P* ≤ 0.05, according to Student's *t*-test.
